# An outbreak of pneumococcal meningitis among older children (≥5 years) and adults after the implementation of an infant vaccination programme with the 13-valent pneumococcal conjugate vaccine in Ghana

**DOI:** 10.1186/s12879-016-1914-3

**Published:** 2016-10-18

**Authors:** Brenda Anna Kwambana-Adams, Franklin Asiedu-Bekoe, Badu Sarkodie, Osei Kuffour Afreh, George Khumalo Kuma, Godfred Owusu-Okyere, Ebenezer Foster-Nyarko, Sally-Ann Ohene, Charles Okot, Archibald Kwame Worwui, Catherine Okoi, Madikay Senghore, Jacob Kweku Otu, Chinelo Ebruke, Richard Bannerman, Kwame Amponsa-Achiano, David Opare, Gemma Kay, Timothy Letsa, Owen Kaluwa, Ebenezer Appiah-Denkyira, Victor Bampoe, Syed M. A. Zaman, Mark J. Pallen, Umberto D’Alessandro, Jason M. Mwenda, Martin Antonio

**Affiliations:** 1 Vaccines and Immunity Theme, The Medical Research Council Unit The Gambia, P.O Box 273, Banjul, Fajara The Gambia; 2 Ghana Health Service, Accra, Ghana; 3 Brong Ahafo Regional Health Directorate, Sunyani, Brong Ahafo Ghana; 4 Regional Hospital Sunyani, Sunyani, Brong Ahafo Region Ghana; 5 National Public Health Reference Laboratory, Ghana Health Service, Accra, Ghana; 6 WHO Country Office Ghana, Accra, Ghana; 7 Microbiology and Infection Unit, Warwick Medical School, Warwick, UK; 8 Ministry of Health, Accra, Ghana; 9 Disease Control and Elimination Theme, Medical Research Council Unit The Gambia, Fajara, The Gambia; 10 London School of Hygiene and Tropical Medicine, London, UK; 11 Institute of Tropical Medicine, Antwerp, Belgium; 12 WHO Regional Office for Africa, Brazzaville, Republic of Congo

**Keywords:** Pneumococcus, Outbreak, Serotype 1, Ghana, Meningitis belt, West Africa, Meningitis, Pneumococcal conjugate vaccine (PCV)

## Abstract

**Background:**

An outbreak of pneumococcal meningitis among non-infant children and adults occurred in the Brong-Ahafo region of Ghana between December 2015 and April 2016 despite the recent nationwide implementation of a vaccination programme for infants with the 13-valent pneumococcal conjugate vaccine (PCV13).

**Methods:**

Cerebrospinal fluid (CSF) specimens were collected from patients with suspected meningitis in the Brong-Ahafo region. CSF specimens were subjected to Gram staining, culture and rapid antigen testing. Quantitative PCR was performed to identify pneumococcus, meningococcus and *Haemophilus influenzae*. Latex agglutination and molecular serotyping were performed on samples. Antibiogram and whole genome sequencing were performed on pneumococcal isolates.

**Results:**

Eight hundred eighty six patients were reported with suspected meningitis in the Brong-Ahafo region during the period of the outbreak. In the epicenter district, the prevalence was as high as 363 suspected cases per 100,000 people. Over 95 % of suspected cases occurred in non-infant children and adults, with a median age of 20 years. Bacterial meningitis was confirmed in just under a quarter of CSF specimens tested. Pneumococcus, meningococcus and Group B Streptococcus accounted for 77 %, 22 % and 1 % of confirmed cases respectively. The vast majority of serotyped pneumococci (80 %) belonged to serotype 1. Most of the pneumococcal isolates tested were susceptible to a broad range of antibiotics, with the exception of two pneumococcal serotype 1 strains that were resistant to both penicillin and trimethoprim-sulfamethoxazole. All sequenced pneumococcal serotype 1 strains belong to Sequence Type (ST) 303 in the hypervirulent ST217 clonal complex.

**Conclusion:**

The occurrence of a pneumococcal serotype 1 meningitis outbreak three years after the introduction of PCV13 is alarming and calls for strengthening of meningitis surveillance and a re-evaluation of the current vaccination programme in high risk countries.

**Electronic supplementary material:**

The online version of this article (doi:10.1186/s12879-016-1914-3) contains supplementary material, which is available to authorized users.

## Background

Acute bacterial meningitis is most commonly caused by *Neisseria meningitidis*, *Streptococcus pneumoniae* and *Haemophilus influenzae* Type b. In sub-Saharan Africa, there is a “meningitis belt” running from Ethiopia to Senegal where there is high seasonal incidence of bacterial meningitis. In West Africa, the highest incidence of bacterial meningitis occurs during the dry season (December to March) [1–3], with incidence rates in epidemics as high as 800 cases per 100,000 people [4, 5].

*N. meningitidis*, the meningococcus, is the leading cause of bacterial meningitis in West Africa after the first year of life, even in non-epidemic periods [1–3, 6, 7]. In the past, most West African meningococcal outbreaks were attributed to serogroup A [8]. However, the MenAfriVac™ vaccine, which protects against meningococcal serogroup A, has been rolled out in several countries in the meningitis belt. In Ghana, MenAfriVac™ vaccination campaigns conducted in 2012 covered the three northern regions within the meningitis belt [4].

Outbreaks of *S. pneumoniae*, the pneumococcus, were previously thought to be restricted to prisons, military camps and among people living in crowded conditions such as refugee camps [9–13]. However, in the early 2000s, large pneumococcal outbreaks were reported in a few West African countries including Ghana [3, 14–16]. A sustained increase in the cases of pneumococcal meningitis was reported in Northern Ghana between 2000 and 2003. The incidence of pneumococcal meningitis rose from ≤5 cases/100,000 people/year in 2000 to 15–26 cases/100,000 people/year at its peak [16]. Pneumococcal meningitis outbreaks were also reported in neighboring countries Burkina Faso, Togo and Niger around the same period [3, 14–16]. These West-African outbreaks have been characterised by high case-fatality rates (up to 40 %) and appear to mimic meningococcal meningitis outbreaks, with peaks in the hot dry season [3, 14, 16]. Pneumococci expressing the serotype 1 capsule dominated the West African pneumococcal meningitis outbreaks. Most of the strains belonged to the clonal complex dominated by Sequence Type (ST)217 [14, 16, 17].

The 13-valent pneumococcal polysaccharide-diphtheria CRM_197_ protein conjugate vaccine (PCV13) now part of the routine immunization programmes in many countries is particularly efficacious in infants. PCVs are thought to elicit mucosal immunity, possibly due to the induction of opsonizing IgA antibodies [18]. Widespread use of PCVs markedly reduces carriage of vaccine serotypes amongst both vaccinated and unvaccinated individuals [19–21]. PCVs are a remarkable public health success as they induce herd immunity and are associated with reductions in invasive pneumococcal disease globally [19–21]. PCV13 was introduced in the Ghana Expanded Programme on Immunisation (EPI) in April 2012.

The Brong Ahafo Region spans the central section of Ghana on the north–south axis and falls outside the meningitis belt, which is restricted to the northern region of the country. The WHO designated Regional Reference Laboratory (RRL) hosted at the Medical Research Council Unit The Gambia coordinates surveillance of invasive bacterial diseases across West Africa. The WHO RRL supported the Ghana Ministry of Health to confirm the causative pathogens and provided technical laboratory support teams at regional and district hospitals in the Brong Ahafo Region for bacteriologic processing of CSF specimens. The aim of this study was to confirm and characterise the pathogens that caused this unusual outbreak in Ghana.

## Methods

### Study area

The Brong-Ahafo Region is one of ten regions in Ghana and has a population of 2.4 million distributed in 27 districts. It has a bi-modal rainfall pattern with the major rainy season occurring between April and July and a minor rainy season between September and October. The dry season runs from November to March.

### Patients

All cases of suspected meningitis in Brong Ahafo Region, which presented at private, mission, district and regional hospitals had a lumbar puncture performed. Suspected meningitis was defined as sudden onset of fever (>38.5 °C) and a combination of any of the following clinical symptoms: reduced level of consciousness, stiff neck, bulging fontanel, fit(s) if aged between six months and five years or partial seizures. When available, the CSF specimens were stored in Trans-isolate (T-I) media. The patients were treated with ceftriaxone or ampicillin.

### Bacteriologic analysis of CSF specimens

CSF samples were initially processed at the health centers or hospitals where they were collected. However, when this was not possible, the processing was performed at the Regional Hospital, Sunyani. The primary method for the detection of pneumococcus, *Haemophilus influenzae* type b, meningococcus serogroups A, C, Y/W was the Pastorex meningitis kit (Biorad, UK), which was used following manufacturer’s instructions.

For culture, 10 μl of un-centrifuged CSF was streaked onto Columbia blood agar (BA) and chocolate agar (CA) plates and incubated at 37 °C in 5 % CO_2_ for 18–24 h (overnight). 1–2 drops of CSF were used to prepare a smear, air-dried and fixed by flooding with 95 % ethanol for 2 min or by passing through the flame for a few seconds. Gram stain was performed following WHO protocol [22].

Following overnight incubation, the CA and BA plates were examined for characteristic growth of pneumococcus, *H. influenzae*, meningococcus and other pathogens. Suspected pneumococcal colonies were confirmed and serotyped as previously described [23]. All suspected meningococcus or *H. influenzae* underwent biochemical confirmation using analytical profile index kits (API NH; Biomerieux, UK). Meningococcal serogroups were assessed by use of the Directigen™ meningitis combo kit (Oxoid Basingstoke, UK) following manufacturer’s instructions. Pneumococcal isolates underwent antibiotic susceptibility testing by the disc and E-test diffusion methods for commonly prescribed antibiotics in the sub-region following CSLI guidelines [24]. Antibiotic activity in the CSF specimens was assessed by the disc diffusion antibiotic bioassay following the method described previously by Driscoll et al., (2012) [25]. All antibiotics were procured from Oxoid (Basingstoke, UK) and E-test strips from Biomerieux, UK.

The MRC Unit The Gambia, molecular microbiology laboratory submits to the external quality assurance programme of the UK National External Quality Assessment Service (http://www.ukneqas.org.uk) and is a World Health Organization (WHO) Regional Reference Laboratory for invasive bacterial pathogens.

### Real time PCR detection and serotyping of pathogens

An aliquot of the CSF specimens was shipped to the WHO RRL hosted at MRCG in dry ice (−80 °C). Species-specific quantitative PCR (qPCR) assays for detection of pneumococcus, meningococcus and *H. influenzae* were conducted using the autolysin gene (*lytA*), the CU, Zn superoxide dismutase gene (*sodC*) and the protein D encoding gene (*hpd*) respectively as previously described [26–28]. *RNaseP* gene assay was performed on all CSF specimens to confirm samples of human origin and the integrity of the CSF specimens. Positivity for each of the targets was deduced using cycle threshold (CT) values. CTs of ≤36 were considered as positive.

### Serogroup and serotype specific qPCR assays

Meningococcal serogrouping and *H. influenzae* serotyping were performed by direct qPCR as previously described [27]. Targets for the mentioned pathogens included *sacB, synD*, *synE*, *synG*, *xcbB*, *synF* genes for serogroups A, B, C, W, X, Y respectively. For *H. influenzae*, the following serotypes were screened: *acB* (Hia), *bcsB* (Hib), *ccsD* (Hic), *dscE* (Hid), *ecsH* (Hie) and *bexD* (Hif).

### *Streptococcus pneumoniae* serotyping

In preparation for nucleic acid extraction for pneumococcal serotyping, 200 μL of CSF was added to 50 μL of TE buffer containing 0.08 g/mL of lysozyme (Sigma-L-6876) and 150U/mL of mutanolysin (Sigma M-9901), and mixture was incubated for one hour at 37 °C. The remaining extraction procedures followed Qiagen DNA Mini kit (Qiagen, UK) manufacturer’s instructions.

Purified DNA extracts were subjected to sequential triplex qPCR assay for detecting 21 pneumococcal capsular serotypes for the African scheme as previously described [29]. CSF specimens with CT values ≤32 were further subjected to conventional multiplex PCRs also described elsewhere [30, 31].

### Whole-genome sequencing

Whole-genome sequencing was performed on nine purified pneumococcal isolates cultured from CSF specimens. All the isolates could not be sequenced due to cost considerations. Sequencing was performed on the illumina Miseq platform following a NexteraX library preparation step. Genomic data was analysed using the nullarbor pipeline (https://github.com/tseemann/nullarbor), which confirmed the species of the isolates, inferred MLST types, reported the presence of resistance genes and extracted a core genome. Draft genome assemblies for the nine outbreak strains were generated using spades (kmers: 21, 33, 55, 77, 99 and 127). To illustrate the phylogenetic relationship of the outbreak ST303 isolates, we included nine archived ST303 draft pneumococcal genome assemblies [32]. The assembled ST303 genomes from the pneumococcal outbreak strains and the draft genomes were compared against the P1031 pneumococcal reference genome; a pneumococcal historical isolate from Ghana [33]. Contigs were mapped against the reference and SNVs were called from the core genome using parsnp [34]. The pneumococcal genome sequences are available on the European Bioinformatics Institute (EBI) database under study accession PRJEB15437 at http://www.ebi.ac.uk/ena/data/view/PRJEB15437.

### Data management

Demographic and clinical data of suspected cases were collected with designed case report forms and entered into Microsoft Excel and an EPI Info database tool. Demographic and clinical data of the cases were extracted into a line list. The Excel data sets from the health facilities were imported and merged into a Microsoft Access database. Pathogen detection, serotyping and serogrouping data from qPCR and other methods were imported and merged with demographic and clinical data in the customized Microsoft Access database. Antibiotic and antibiogram data were also entered into the customized Microsoft Access database. Data analysis was carried out using GraphPad Prism, R statistics tool (version 3.2.4) for Windows and Microsoft Excel. The maps were generated using QGIS v1.0 software using Geographical Position System (GPS) coordinates from the US Geographical Survey (USGS) https://www.usgs.gov.

## Results

**Fig. 1:**
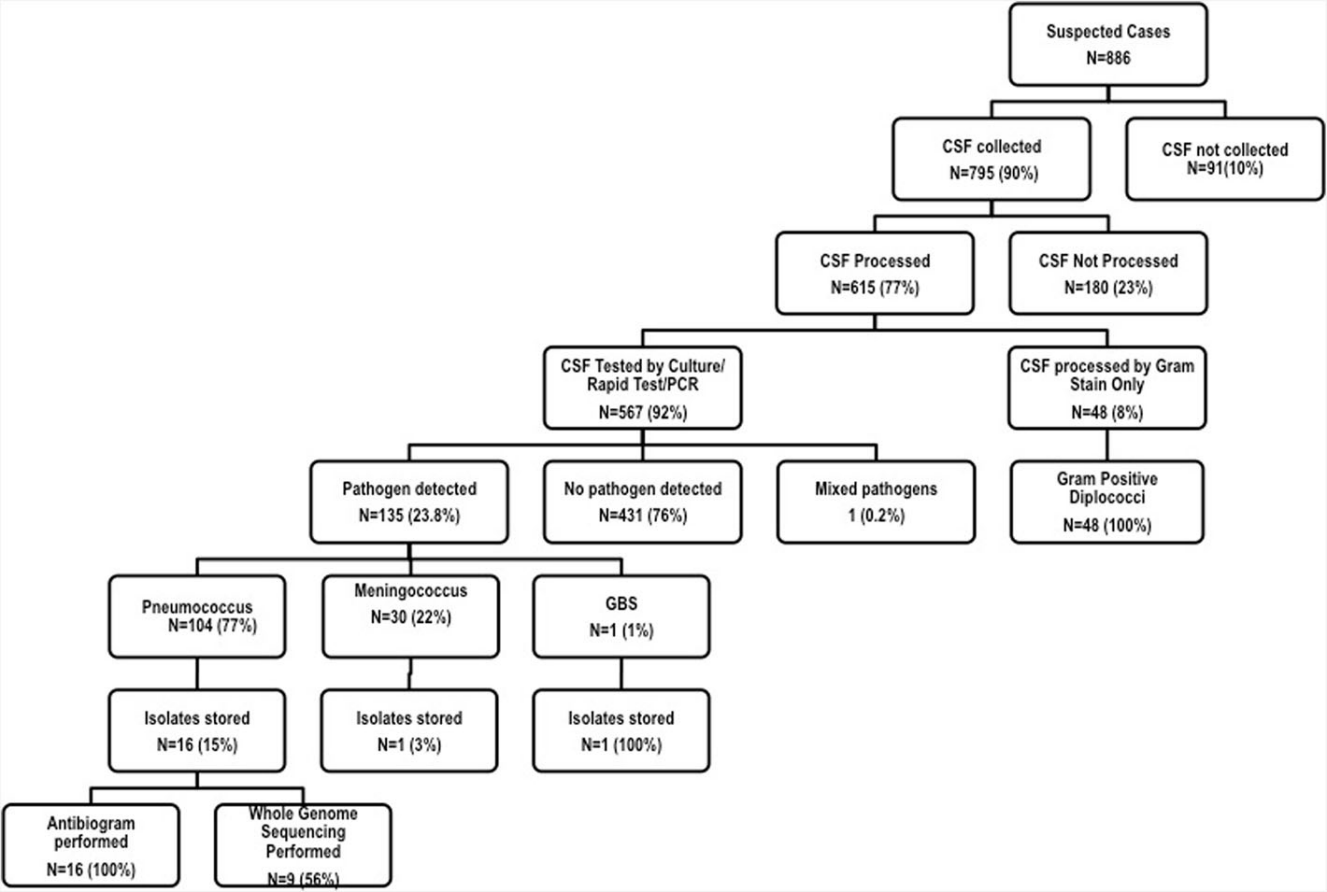
Summary of CSF specimen collection and processing

The nationwide weekly incidence of meningitis rose from <5 cases/100,000 people in the 49th week of 2015 to 350 cases/100,000 people in the 7th week of 2016. Between 2 December 2015 (49th week) and 26 February 2016 (9th week), there were 886 suspected meningitis cases reported. A flow chart of the CSF specimens collected, tested and the outcomes are summarised in Fig. 1. Overall, 64 % (567/886) of CSF specimens were tested by rapid test, culture and/or qPCR. Meningitis was confirmed in 23.8 % (135/567). Pneumococcus, meningococcus and GBS accounted for 77 % (104/135), 22 % (30/135) and 1 % (1/135) of the confirmed cases of meningitis. One CSF was positive for both meningococcus and pneumococcus, an unusual finding probably due to contamination (Fig. 1).

The characteristics of the patients with suspected and confirmed meningitis are summarised in Table 1. The patients’ median age was 20 years (IQR 13 and IQR 32) and the range was 9 months to 98 years. 15 to 29 year olds accounted for 40 % of the suspected and confirmed cases. Less than 5 % of the suspected and confirmed cases were among children less than 4 years old who would likely have received PCV13 (Table 1).

**Table 1:** Patient characteristics and outcome

Characteristic	Category	Suspected Cases *n* (%)	Confirmed Cases *n* (%)	*S. Pneumoniae n* (%)	*N. Meningitidis n* (%)	*Group B Streptococcus n* (%)	*Mixed pathogen*
Gender	Female	458 (51.6)	55 (40.4)	43 (41.3)	10 (33.3)	1 (10)	1 (10)
	Male	348 (39.2)	54 (39.7)	42 (40.3)	12 (40.0)	0 (0)	0 (0)
	Unknown	80 (9.0)	27 (19.8)	19 (18.2)	8 (26.6)	0 (0)	0 (0)
Age	<1	11 (1.2)	2 (1.4)	2 (1.9)	0 (0)	0 (0)	0 (0)
	1–4	26 (2.9)	4 (2.9)	3 (2.8)	1 (3.3)	0 (0)	0 (0)
	5–14	174 (19.6)	35 (25.7)	30 (28.8)	4 (13.3)	1 (100)	0 (0)
	15–29	354 (39.9)	44 (32.3)	31 (29.8)	12 (4)	0 (0)	1 (100)
	30–59	203 (22.9)	19 (13.9)	16 (15.3)	3 (10)	0 (0)	0 (0)
	>60	37 (4.1)	5 (3.6)	3 (2.8)	2 (6.6)	0 (0)	0 (0)
	Unknown	81 (9.1)	27 (19.8)	19 (18.2)	8 (26.6)	0 (0)	0 (0)
	January	267 (30.1)	74 (54.4)	58 (55.7)	15 (5)	0 (0)	1 (100)
	February	499 (56.32)	30 (22.0)	22 (21.1)	7 (23.3)	1 (100)	0 (0)
	Unknown	100 (11.28)	27 (19.8)	19 (18.2)	8 (26.6)	0 (0)	0 (0)
Outcome	Alive	711 (80.2)	76 (55.8)	60 (57.6)	14 (46.6)	1 (10)	1 (100)
	Dead	75 (8.4)	33 (24.2)	25 (24.0)	8 (26.6)	0 (0)	0 (0)
	Unknown	100 (11.2)	27 (19.8)	19 (18.2)	8 (26.6)	0 (0)	0 (0)

**Fig. 2:**
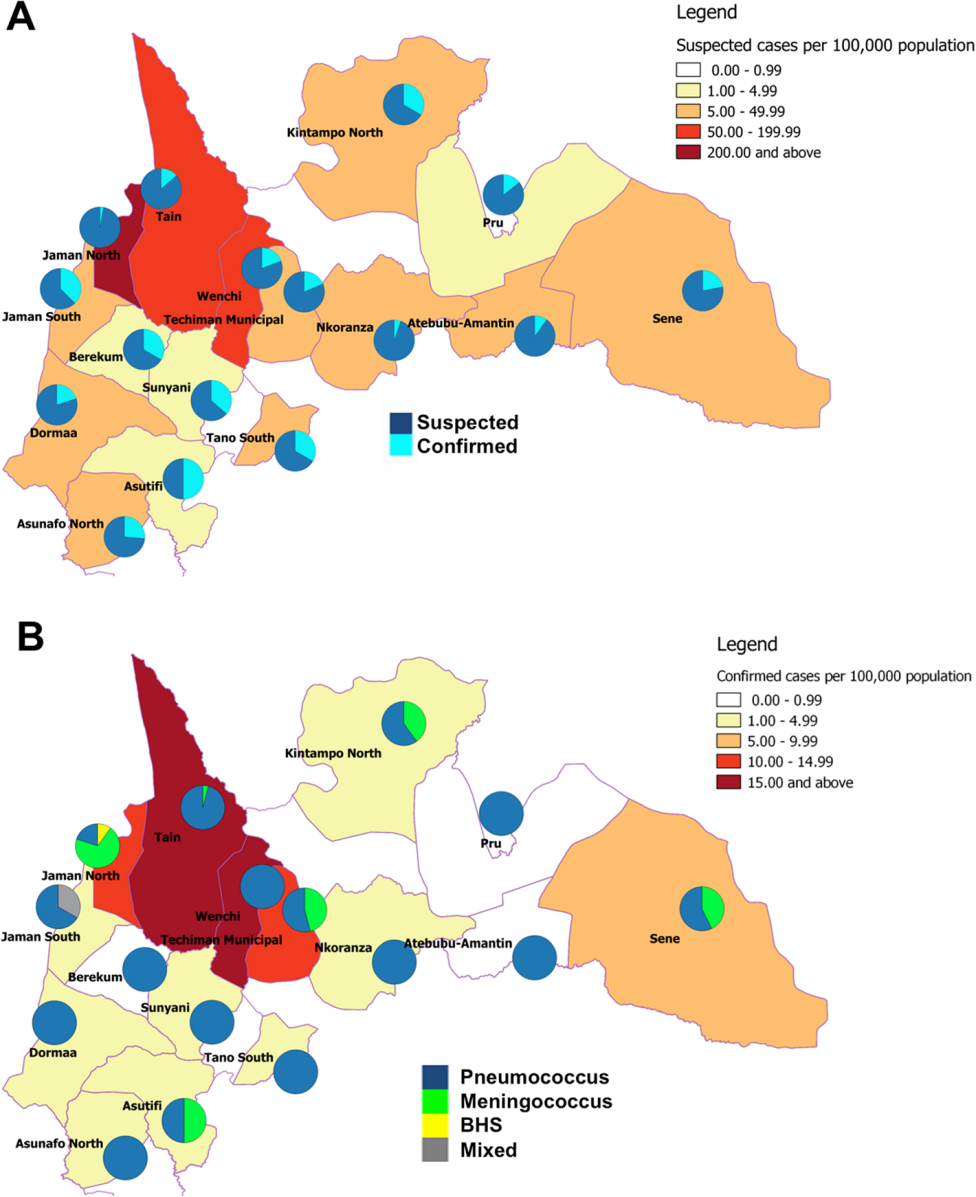
The geographical distribution of the suspected and confirmed meningitis cases in Brong Ahafo region. The districts are shaded by suspected meningitis per 100,000 population and the pie charts representing the proportion of confirmed cases (**a**). The districts are shaded by the confirmed cases per 100,000 population and the pie charts represent the distribution of pathogens (**b**). The maps were generated using QGIS v1.0 software using Geographical Position System (GPS) coordinates from the US Geographical Survey (USGS) https://www.usgs.gov

Although suspected meningitis patients were reported across the whole region, the epicenter of the outbreak was in four contiguous districts; Jaman North, Tain, Wenchi and Techiman municipal (Fig. 2a). There were 363 suspected meningitis cases per 100,000 people in Jaman North, more than double that reported in Tain with 156 per 100,000 people. Although Jaman North had the highest rate of suspected cases, Tain and Wenchi had the highest rates of confirmed cases. There were 25, 20, 11 and 10 confirmed cases per 100,000 people in Tain, Wenchi, Jaman North and Techiman Municipal, respectively (Fig. 2a/b). The number of confirmed cases declined after the 6th week of 2016, but those of suspected cases increased up to the 8th week of 2016 as shown in Additional file 1. The meningitis outbreak peaked at different weeks across the hardest hit districts (Additional file 1). Tain district crossed the epidemic threshold of 10 suspected cases per 100,000 population in week 53 of 2015 whereas Wenchi and Jaman North district crossed the epidemic threshold in weeks 3 and 6 of 2016 respectively (Additional file 1).

**Fig. 3:**
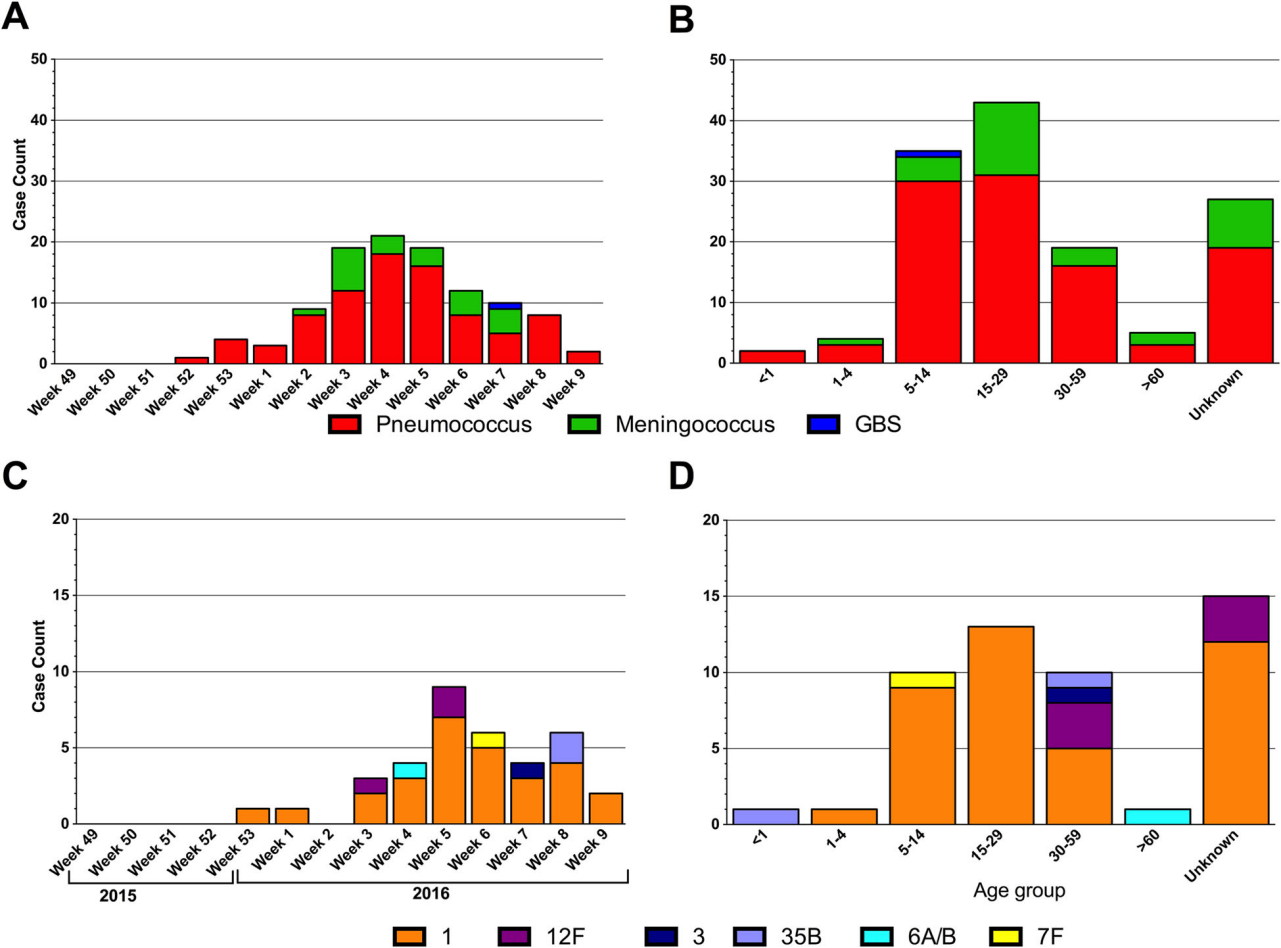
The distribution of pathogens by week and age. The distribution of pathogens by week (**a**) and age (**b**). The distribution of pneumococcal serotypes by week (**c**) and age (**d**)

Pneumococcus was the sole or leading pathogen across all districts with the exception of Jaman North district where meningococcus was the dominant pathogen accounting for 63 % (7/11) of confirmed cases (Fig. 2b). Pneumococcus was the leading pathogen from the 52nd week of 2015 to the 9th week of 2016 (Fig. 3a). Meningococcus was detected between weeks 2 and 7 of 2015. GBS was cultured from a CSF specimen in week 7 of 2015 (Fig. 3a).

The molecular and latex serotyping results by age group are summarised in Table 2. A tenth (10/104) of the pneumococci detected by PCR were either non-typeable by PCR or had a High Ct for molecular typing. Forty-five pneumococci could not be typed as the isolate and/or CSF was not available for serotyping. Serotype 1 accounted for 78 % (38/49) of the serotyped pneumococci. The distribution of the other serotypes was 12 % (6/49) serotype 12 F, 4 % (2/49) serotype 35B, 2 % (1/49) serotype of each, serotype 6A/6B, serotype 7 F and serotype 3. The only infant (10.8 month old) with confirmed pneumococcal meningitis had a serotype 35B infection, a non-PCV13 serotype. Serotype 1 was the only pneumococcal serotype detected among patients between four and 29 years old (Fig. 3d). In contrast, serotype 12 F, also a non-PCV13 serotype, was confirmed among cases aged ≥30 years. The serotype 12 F pneumococcal strains were detected across five districts including Tain in weeks 3 and 5 of 2015. The two serotype 35B strains were detected in Dormaa in week 8 of 2016 (Fig. 3c). Of the 20 meningococci that could be serotyped, 80 % (16/20) were serogroup W, 5 % (1/20) was a serogroup C and 15 % (3/20) could not be grouped by the real time PCR serotyping panel that includes serogroups A, B, C, W, X and Y.

**Table 2:** Distribution of pneumococcal serotypes which caused meningitis by age group

Age Group	1	12 F	3	35B	6A/6B	7 F	^a^High Ct value	^b^Nontypeable	^c^Not serotyped	Total
<1	0	0	0	1	0	0	0	1	0	2
1–4	1	0	0	0	0	0	0	1	1	3
5–14	8	0	0	0	0	1	0	1	20	30
15–29	12	0	0	0	0	0	0	2	17	31
30–59	5	3	1	1	0	0	0	0	6	16
>60	0	0	0	0	1	0	1	0	1	3
Unknown	12	3	0	0	0	0	2	2	0	19
Grand Total	38	6	1	2	1	1	3	7	45	104

^a^These CSF had a Ct value >32 and <36 for pneumococcal detection which is above the threshold for molecular serotyping

^b^The pneumococci could not be serotyped using molecular techniques which detect a limited panel of serotypes

^c^The CSF specimens and isolates were not available for serotyping

A tenth (75/786) of the patients with suspected meningitis and a known outcome died. Among the confirmed cases, death was reported among 24 % (33/135) of the patients (Table 1). Amongst the deaths, the average length of time before reporting was 1.7 days and the average length of stay between admission and death was 2.3 days. The case fatality rates for confirmed meningococcal and pneumococcal meningitis were 36 % (8/22) and 29 % (25/85) respectively. The eight-year-old child with GBS meningitis survived.

Pneumococcal and meningococcal isolates were confirmed at the WHO RRL and underwent antibiotic susceptibility testing by disc diffusion and E-test [24]. Seventeen pneumococcal isolates from CSF were available for antimicrobial susceptibility testing by disc diffusion and E-test methods for commonly prescribed antibiotics in the sub-region following CSLI guidelines [24]. The antibiogram of the isolates, serotype and the basic patient characteristics are summarised in Additional file 2. All the pneumococcal isolates tested were fully susceptible to ceftriaxone, vancomycin, chloramphenicol, clindamycin, erythromycin and rifampin. In contrast, 76 % (13/17) of the strains were resistant or had intermediate resistance to trimethoprim-sulfamethoxazole. Over 80 % (14/17) of the isolates were resistant to tetracycline. Resistance to penicillin was found in two isolates, both serotype 1 strains. These two serotype 1 strains were also resistant to trimethoprim-sulfamethoxazole and tetracycline. Both serotype 35B strains were fully susceptible to all the antibiotics screened. Antimicrobial activity in the CSF specimens was assessed by the disc diffusion antibiotic bioassay. Antimicrobial activity was detected in 1.5 % (5/325) of the CSF specimens tested. A pathogen was detected in 2 of the 5 CSF specimens with antimicrobial activity.

**Fig. 4:**
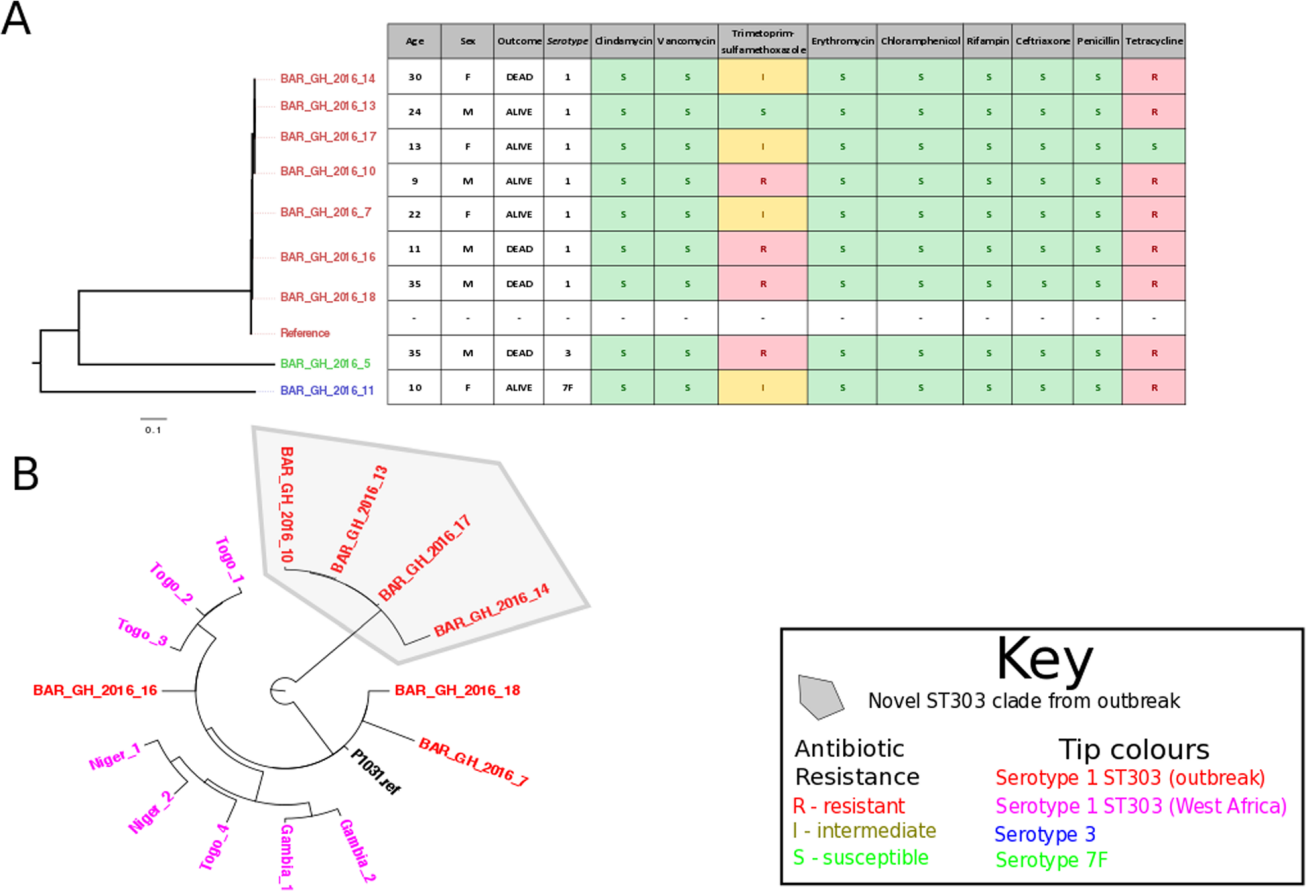
Phylogenetic analysis of outbreak strains. Phylogenetic tree of outbreak strains with antibiogram and patient data (**a**). Phylogenetic analysis of serotype 1 outbreak ST303 strains and historical West African ST303 strains. A novel clade of ST303 serotype distinct from the historic West African ST303 strains is evident (**b**)

Whole genome sequencing was performed on nine randomly selected pneumococcal isolates. The predominant lineage was ST303 (*n* = 7) found among serotype 1 isolates. The serotype 3 and 7 F strains belonged to ST700 and ST2833 lineages respectively. The phylogenetic tree of our outbreak strains showed that the ST303 isolates formed two separate clusters (Fig. 4a). To illustrate the diversity of ST303 within the outbreak, the phylogeny was reconstructed from the ST303 outbreak strains and other previously sequenced ST303 pneumococcal genomes (Fig. 4b). Four outbreak isolates (BAR_GH_2016_10, 13, 14 and 17) clustered on a clade characterized by a long branch from the closest relatives and short branches within the clade. The average pairwise distance between strains on this branch was 18 core genome SNVs. BAR_GH_2016_16 bore similarity to a cluster of ST303 previously isolated from Togo, a neighboring country, east of Ghana. BAR_GH_2016_7 and 18 presented as outgroups in our phylogeny without any marked identity to other isolates.

## Discussion

To our knowledge, this is the first large pneumococcal meningitis outbreak to occur in Ghana since the early 2000s and the first report of a pneumococcal meningitis outbreak outside of the Northern regions of Ghana. [16]. Between 2004 and 2013, there were on average 600 cases of suspected and confirmed meningitis reported per year in Ghana [35]. However, at least 886 cases of suspected meningitis, probable and confirmed meningitis had been reported in Brong Ahafo Region alone by the 9th week of 2016. Although pneumococcal meningitis affected individuals of all ages, nearly 60 % of the cases were among individuals between 5 and 29 years old. Nearly 80 % of the confirmed pneumococcal meningitis cases were attributed to serotype 1. Whole genome sequencing of pneumococcal isolates showed that the serotype 1 strains from the outbreak belonged to the hypervirulent ST217 clonal complex [14, 33]. Although the increasingly important role of pneumococcus in meningitis outbreaks has been reported previously [14, 16, 17], the occurrence of a pneumococcal meningitis outbreak outside the meningitis belt post PCV13 introduction is alarming and highlights the need for vigilant monitoring of pneumococcal invasive disease in Ghana.

Implemented pneumococcal vaccination strategies in Ghana and across Africa almost exclusively target infants. The huge burden of invasive pneumococcal disease among children less than 5 years old globally, together with cost considerations and the lack of data on the burden of disease in other age categories, probably led to policies that protect the youngest and most vulnerable. PCV13 was introduced in Ghana in 2012 and includes serotype 1, which is probably why less than 5 % of pneumococcal meningitis cases were among children less than five years old. In northern Ghana, between 1998 and 2003 prior to the introduction of PCV into the expanded programme of immunization (EPI), the highest incidence of pneumococcal meningitis was found among infants with an annual incidence of 43 cases per 100,000 population [16]. Likewise in neighboring Burkina Faso and Togo between 2002 and 2006, 36 % of pneumococcal meningitis cases were among children less than 5 years old prior to the introduction of pneumococcal conjugate vaccines [3].

PCVs induce herd immunity, markedly reducing vaccine serotype carriage and invasive pneumococcal disease amongst both vaccinated and unvaccinated individuals [36]. However, the high prevalence of pneumococcal serotype 1 meningitis among older children (≥5 years) and adults heralds the need to revisit the current policy and to include all age groups in high-risk populations. Kenya has recorded success with an extensive catch up campaign with PCV-10 for all children less than five years old; there was a 66 % decline in carriage of vaccine serotypes in individuals older than 5 years [37]. If the cost of rolling out PCV13 across all age groups is considered an obstacle, the possibility of implementing a pneumococcal serotype 1 monovalent conjugate vaccine following the model used for meningococcus could be explored. However, it is important to note that the national PCV13 coverage in Ghana was only 41 % and 68 % in 2012 and 2013, respectively, which may be inadequate to induce herd immunity. Although PCV13 coverage rose to 85 % in 2014, maintenance of this coverage and close monitoring of vaccine serotype carriage and disease are needed. Furthermore, the occurrence of serotype 12 F and 35B meningitis, both non-PCV13 serotypes, is also a cause for concern. Serotype 12 F in particular is emerging as an important cause of invasive pneumococcal disease with the propensity to cause outbreaks [38–40].

Leimkugel and colleagues reported that ST303 accounted for 55 % of the serotype 1 strains responsible for a meningitis outbreak in northern Ghana in 2001–2003. All the other serotype 1 strains were clonally related and belonged to the ST217 clonal complex [16]. Likewise, closely related serotype 1 lineages belonging to the ST217 clonal complex were the predominant strains in the highly lethal meningitis epidemic between 2003 and 2004 in Burkina Faso, which borders northern Ghana [14]. All seven serotype 1 strains sequenced in the 2015–2016 Ghana meningitis outbreak belonged to ST303 and at least four are very likely to be the product of a recent clonal expansion within ST303. These strains, which belong to a unique clade, are likely to have diverged from the other previously sequenced ST303 lineages, including strain P1031 from a previous meningitis outbreak in Ghana [16].

All the pneumococcal isolates were fully susceptible to chloramphenicol and ceftriaxone. The recommended treatment for pneumococcal meningitis is vancomycin and a third generation cephalosporin (ceftriaxone and cefotaxime) or rifampin. Similarly, the recommended antimicrobial therapy for meningococcal meningitis is ceftriaxone or cefotaxime although penicillin G, ampicillin and chloramphenicol are also effective treatments [41]. It is reassuring that all the pneumococcal isolates tested were fully susceptible to ceftriaxone and chloramphenicol. Two isolates were resistant to three antibiotics, penicillin G, trimethoprim-sulfamethoxazole and tetracycline. The only meningococcal isolate available for antimicrobial susceptibility testing showed intermediate resistance to chloramphenicol. However, more isolates would have to be tested to confirm the prevalence of reduced susceptibility within the population.

This study has shown that pneumococcal outbreaks caused by closely related strains can reoccur within West Africa. Although the factors that drive pneumococcal meningitis outbreaks are not well understood, it is likely that environmental factors play an important role. As with meningococcal meningitis outbreaks, the highest incidence occurs during the dry season (December to March) [1–3] in West African countries. Greenwood has suggested that the hot dry conditions damage the mucosal defences increasing vulnerability to invasive pneumococcal and meningococcal disease [42]. Although this is a plausible contributing factor, there are likely to be other environmental and biological determinants of pneumococcal meningitis outbreaks that need to be investigated.

## Conclusions

The results reported here are of great public health importance and summarised as follows: 1) Pneumococcus continues to be of increasing importance in meningitis outbreaks, even outside the meningitis belt; 2) In a post-PCV13 era, pneumococcal serotype 1 still has the propensity to cause large outbreak; 3) Non-vaccine serotypes such as serotype 12 F can be important causes of meningitis during outbreaks. The reoccurrence of pneumococcal serotype 1meningitis outbreaks in the sub-region highlights the need for a monovalent vaccine targeted to older non-infant children and adults in high-risk populations. Clear guidelines on the prevention, control and treatment of pneumococcal meningitis outbreaks need to be developed for West Africa. Meningitis surveillance and monitoring is needed even in regions that do not typically experience outbreaks as well as a careful review of the geographical areas previously considered to be at high risk for meningitis outbreaks within the West African sub-region.

## Additional files

Additional file 1: The weekly distribution of suspected meningitis cases in the hardest hit districts. (A) Jaman North District, (B) Tain District, (C) Techiman Municipal District and (D) Wenchi District. (JPG 609 kb)
Additional file 2: Patient characteristics and corresponding *S. pneumoniae* isolates and their antibiotic susceptibility patterns. (DOCX 15 kb)
