# Supplementary figures and images for: An outbreak of pneumococcal meningitis among older children (≥5 years) and adults after the implementation of an infant vaccination programme with the 13-valent pneumococcal conjugate vaccine in Ghana
Source: BMC Infect Dis. 2016 Oct 18;16:575. doi: 10.1186/s12879-016-1914-3 (PMC5070171; doi:10.1186/s12879-016-1914-3)

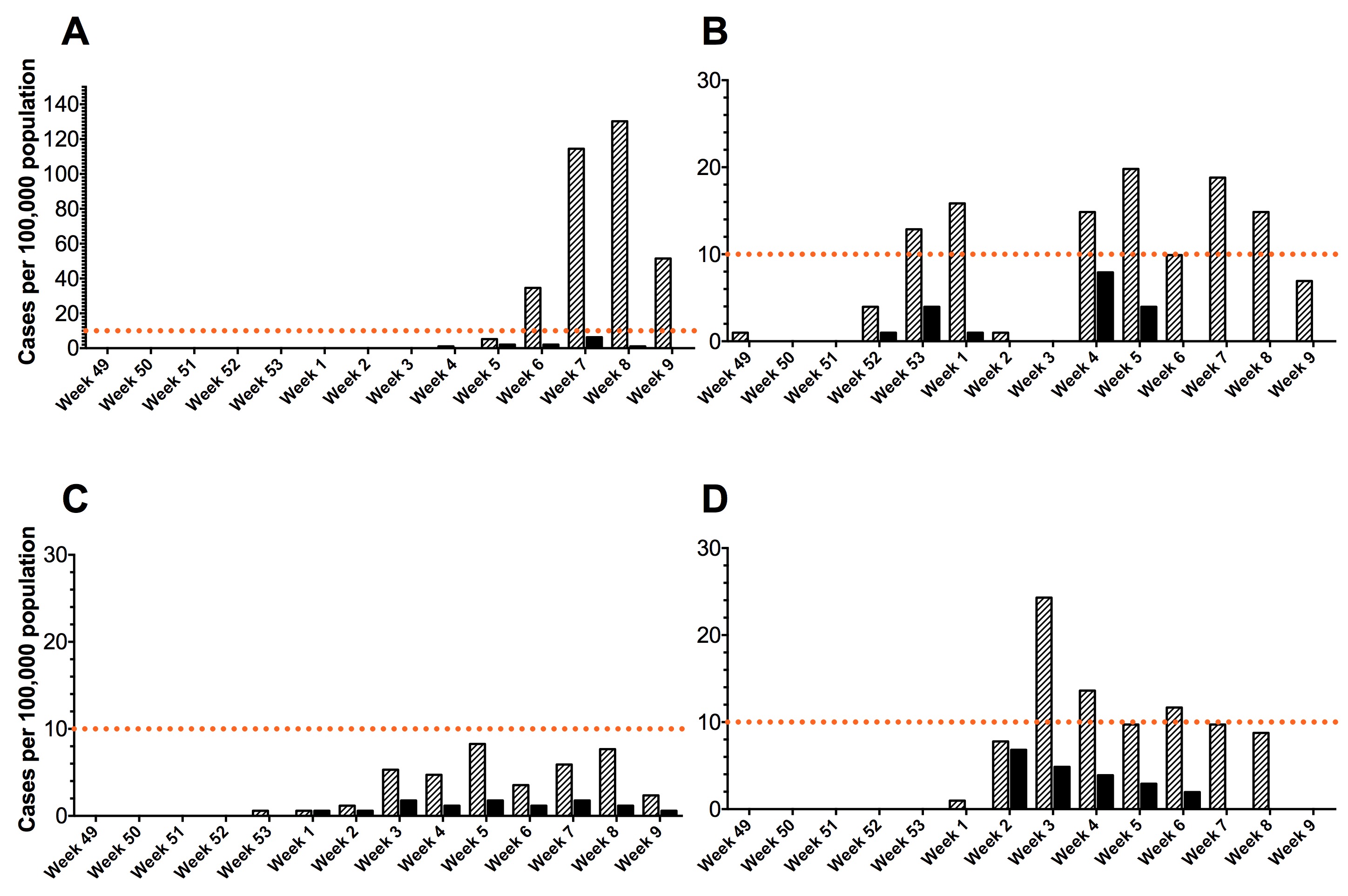

Supplement: Additional file 1: — The weekly distribution of suspected meningitis cases in the hardest hit districts. (A) Jaman North District, (B) Tain District, (C) Techiman Municipal District and (D) Wenchi District. (JPG 609 kb) [file 12879_2016_1914_MOESM1_ESM.jpg]
