# Supplementary material for: An outbreak of pneumococcal meningitis among older children (≥5 years) and adults after the implementation of an infant vaccination programme with the 13-valent pneumococcal conjugate vaccine in Ghana
Source: BMC Infect Dis. 2016 Oct 18;16:575. doi: 10.1186/s12879-016-1914-3 (PMC5070171; doi:10.1186/s12879-016-1914-3)
Supplement: Additional file 2: — Patient characteristics and corresponding S. pneumoniae isolates and their antibiotic susceptibility patterns. (DOCX 15 kb) [file 12879_2016_1914_MOESM2_ESM.docx]

**Additional file 2. Patient characteristics and corresponding *S. pneumoniae* isolates and their antibiotic susceptibility patterns**

| **Isolate name** | **Age range(years)** | **Sex** | **Outcome** | **Serotype** | **Cli** | **Van** | **SXT** | **Ery** | **Chl** | **Rif** | **Cef** | **Pen** | **Tet** |
| --- | --- | --- | --- | --- | --- | --- | --- | --- | --- | --- | --- | --- | --- |
| BAR_GH_2016_1 | 15-29 | M | DEAD | 1 | **S** | **S** | **S** | **S** | **S** | **S** | **S** | **S** | **R** |
| BAR_GH_2016_2 | <1 | M | ALIVE | 35B | **S** | **S** | **S** | **S** | **S** | **S** | **S** | **S** | **S** |
| BAR_GH_2016_3 | 30-59 | F | DEAD | 35B | **S** | **S** | **S** | **S** | **S** | **S** | **S** | **S** | **S** |
| BAR_GH_2016_5 | 30-59 | M | DEAD | 3 | **S** | **S** | **R** | **S** | **S** | **S** | **S** | **S** | **R** |
| BAR_GH_2016_6 | 15-29 | F | ALIVE | 1 | **S** | **S** | **I** | **S** | **S** | **S** | **S** | **S** | **R** |
| BAR_GH_2016_7 | 15-29 | F | ALIVE | 1 | **S** | **S** | **I** | **S** | **S** | **S** | **S** | **S** | **R** |
| BAR_GH_2016_8 | 15-29 | F | ALIVE | 1 | **S** | **S** | **R** | **S** | **S** | **S** | **S** | **R** | **R** |
| BAR_GH_2016_9 | 5-14 | F | ALIVE | 1 | **S** | **S** | **R** | **S** | **S** | **S** | **S** | **S** | **R** |
| BAR_GH_2016_10 | 5-14 | M | ALIVE | 1 | **S** | **S** | **R** | **S** | **S** | **S** | **S** | **S** | **R** |
| BAR_GH_2016_11 | 5-14 | F | ALIVE | 7F | **S** | **S** | **I** | **S** | **S** | **S** | **S** | **S** | **R** |
| BAR_GH_2016_12 | 15-29 | F | DEAD | 1 | **S** | **S** | **R** | **S** | **S** | **S** | **S** | **R** | **R** |
| BAR_GH_2016_13 | 15-29 | M | ALIVE | 1 | **S** | **S** | **S** | **S** | **S** | **S** | **S** | **S** | **R** |
| BAR_GH_2016_14 | 30-59 | F | DEAD | 1 | **S** | **S** | **I** | **S** | **S** | **S** | **S** | **S** | **R** |
| BAR_GH_2016_15 | 15-29 | M | ALIVE | 1 | **S** | **S** | **I** | **S** | **S** | **S** | **S** | **S** | **R** |
| BAR_GH_2016_16 | 5-14 | M | DEAD | 1 | **S** | **S** | **R** | **S** | **S** | **S** | **S** | **S** | **R** |
| BAR_GH_2016_17 | 5-14 | F | ALIVE | 1 | **S** | **S** | **I** | **S** | **S** | **S** | **S** | **S** | **S** |
| BAR_GH_2016_18 | 30-59 | M | DEAD | 1 | **S** | **S** | **R** | **S** | **S** | **S** | **S** | **S** | **R** |

**S=Sensitive I=Intermediate Resistance R=Resistant**

**Cli=clindamycin, Van=vancomycin, Sxt=Trimethoprim-sulfamethoxazole, Ery=erythromycin, Chl=chloramphenicol, Rif=rifampin, Cef=Ceftriaxone, Pen=penicillin, Tet=Tetracycline.**

***Resistance and intermediate resistance were confirmed by E-test**
